# Atomic force microscopy—A tool for structural and translational DNA research

**DOI:** 10.1063/5.0054294

**Published:** 2021-07-09

**Authors:** Kavit H. S. Main, James I. Provan, Philip J. Haynes, Geoffrey Wells, John A. Hartley, Alice L. B. Pyne

**Affiliations:** 1 UCL Cancer Institute, University College London, London WC1E 6DD, United Kingdom; 2 London Centre for Nanotechnology, University College London, London WC1H 0AH, United Kingdom; 3 Institute of Molecular, Cell, and Systems Biology, University of Glasgow, Glasgow G12 8QQ, United Kingdom; 4 Molecular Science Research Hub, Department of Chemistry, Imperial College London, London W12 0BZ, United Kingdom; 5 UCL School of Pharmacy, University College London, London WC1N 1AX, United Kingdom; 6 Department of Materials Science and Engineering, University of Sheffield, Sheffield S1 3JD, United Kingdom

## Abstract

Atomic force microscopy (AFM) is a powerful imaging technique that allows for structural characterization of single biomolecules with nanoscale resolution. AFM has a unique capability to image biological molecules in their native states under physiological conditions without the need for labeling or averaging. DNA has been extensively imaged with AFM from early single-molecule studies of conformational diversity in plasmids, to recent examinations of intramolecular variation between groove depths within an individual DNA molecule. The ability to image dynamic biological interactions *in situ* has also allowed for the interaction of various proteins and therapeutic ligands with DNA to be evaluated—providing insights into structural assembly, flexibility, and movement. This review provides an overview of how innovation and optimization in AFM imaging have advanced our understanding of DNA structure, mechanics, and interactions. These include studies of the secondary and tertiary structure of DNA, including how these are affected by its interactions with proteins. The broader role of AFM as a tool in translational cancer research is also explored through its use in imaging DNA with key chemotherapeutic ligands, including those currently employed in clinical practice.

## INTRODUCTION

**FIG. 1.**
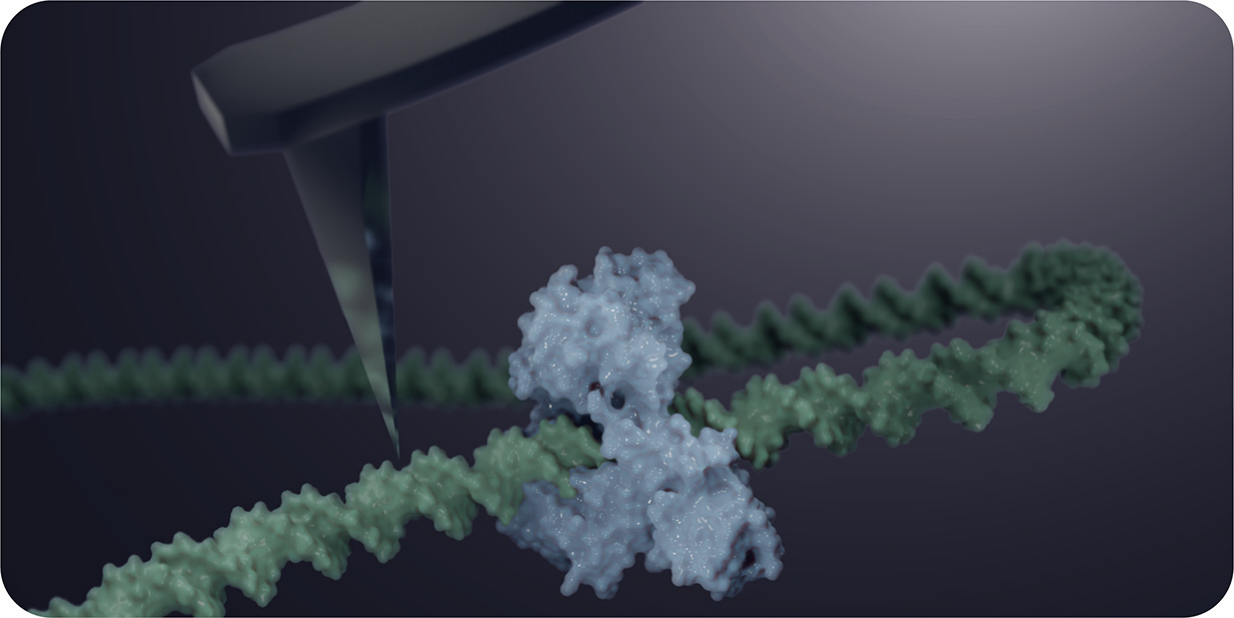
An illustration depicting an AFM cantilever-tip probing a DNA-protein complex in fluid.

Since the seminal crystallography work of Franklin and Gosling^1^ revealed the double helical structure of DNA, characterization of the heterogeneous polymeric structures of DNA has been carried out using a suite of biophysical techniques including x-ray crystallography,^2–4^ electron microscopy,^5–9^ nuclear magnetic resonance (NMR),^10–12^ Förster resonance energy transfer (FRET),^13–16^ and optical and magnetic tweezers.^17–20^ However, limitations in spatial resolution without the requirement for ensemble averaging or labeling have limited the scope for high-resolution studies of the structure of DNA on flexible, individual molecules. It is here that high-resolution AFM can contribute to structural studies of DNA. Namely, AFM can simultaneously probe the flexibility and mechanics of DNA molecules and their local helical structure (Fig. 1).

Atomic force microscopy (AFM) is a technique in the diverse scanning probe microscopy (SPM) family. AFM was first proposed by Binnig *et al.*,^21^ and within 18 months, the technique was used to accomplish atomic-scale imaging of crystalline surfaces.^22^ AFM is a force-based SPM technique, which reconstructs an image of the topography of a sample. Importantly, this facilitates the physical analysis of samples without additional staining or labeling processes. A topographic image is generated by the scanning of a sharp nanometer-sized probe (grown or etched on the free end of a cantilever), which “feels” the contours of the sample surface, analogous to the tactile reading of braille. The topographic image is built up line-by-line by raster-scanning using a piezoelectric scanner to move either the sample or cantilever, depending on the microscope setup. As the tip moves over the sample, the contours of the sample topography induce deflection of the tip and therefore bending of the cantilever. Typically, this bending is measured using the optical lever method where a laser beam is irradiated onto the end of the cantilever and reflected into a photodiode.^23^ As the tip and sample interact, bending of the cantilever results in deflection of the cantilever and displacement of the laser on the photodetector, which is measured as a change in voltage. This change in voltage is used as an input to the feedback loop of the AFM. In the simplest implementation of AFM, constant height contact mode, this change in voltage is converted to a measurement of height that ultimately generates a 3D topographic image of the underlying sample surface.

The high signal-to-noise ratio of the atomic force microscope enables single-molecule imaging with nanometer lateral resolution in solution without ensemble averaging or sample labeling. Shortly after its initial development, AFM was used to generate topographic images of crystalline and biological specimens under aqueous conditions.^24^ The formative study by Drake and colleagues utilized human blood-clotting proteins fibrinogen and thrombin to demonstrate that AFM could image dynamic biological phenomena in real-time, under a physiologically relevant solution, at room temperature.^24^ Progress in AFM development for imaging biomolecules in fluid followed rapidly.^25^ AFM has since been deployed for valuable insight into biological phenomena through single-molecule visualization, from transcription,^26^ to capturing the dynamic movement of myosin walking on actin filaments,^27^ to observing the activity of CRISPR-Cas9 in real-time.^28^

Studies of the structure of DNA and its dynamic interactions with proteins are ideally suited to AFM, due to the long flexible nature and nanometer radius of DNA, which results in a dynamic, conformationally variable range of structures. Several early studies in imaging DNA by AFM developed initial insights into problems of DNA deposition.^29–32^ Gradually, the experimental limitations of AFM of DNA in solution at the time were identified, notably a lack of resolution and reproducibility.^33–38^ Many studies of DNA-protein interactions to date have therefore been performed in air, as a way to simplify deposition and increase reproducibility.^39–43^

AFM has arguably come of age as an accessible method to image single biomolecules and is now routinely used to visualize dynamic processes in real time.^27,44,45^ The technologies underpinning AFM have matured sufficiently to enable routine high-resolution imaging of the nucleic acid structure on a single molecule in a hydrated, uncoated, and dynamic state^46–48^ Notably, these include the development of PeakForce tapping (PFT) mode AFM;^49^ the optimization of smaller or more stable cantilevers;^50,51^ the fine tuning of imaging variables and immobilization strategies.^47,52–54^ In this review, we discuss developments in AFM, which have enabled high-resolution single-molecule imaging of DNA. This includes the range of sample preparation methods and AFM modes used for DNA imaging and how these have been optimized to achieve high resolution, repeatable imaging. We extend this to discuss how improvements in the spatial resolution of AFM has provided new insight into fundamental biological mechanisms, and how these improvements may be applied to the field of translational cancer research. Owing to the large range of applications of the technique, key examples have been chosen to represent the scope of bio-AFM imaging of DNA and DNA–protein–ligand interactions.

## IMMOBILIZATION METHODS FOR AFM IMAGING OF DNA AND DNA-PROTEIN COMPLEXES

Sample preparation is a fundamental part of AFM owing to the mechanical interaction between the tip and sample during operation of the microscope. The sample preparation for AFM studies of DNA and its interactions is relatively simple, requiring the molecule(s) of interest to be adsorbed onto a flat substrate in a stable conformation. It is intuitive that an invasive method of microscopy, capable of imaging the dynamic states of biomolecules, may at times suffer from a lack of resolution resulting from this dynamism. Indeed, AFM of DNA in solution is a fine balancing act between the degree of DNA adsorption (strongly vs loosely adsorbed, as controlled by the immobilization method), imaging parameters (tip-sample interaction forces, scan times, and resolution), and secondary experimental factors that can modulate both. For example, observing live protein-DNA interactions may require potentially sub-optimal conditions for imaging quality, due to the chemical composition of the imaging buffer required for protein solubility and activity.

From the earliest studies of DNA using AFM, the principal substrate used to immobilize DNA has been muscovite mica.^32,55,56^ Mica is a silicate mineral consisting of weakly interacting planes that cleave to the thickness of a single atom. The resultant cleaved surface is atomically flat over mm^2^ length scales, which allows extremely precise and consistent topographical measurements across a sample.^57,58^ However, mica and DNA both have a net negative charge under pH-neutral conditions, resulting in significant electrostatic repulsion. To achieve surface immobilization of DNA for imaging by AFM, additional surface modifications are required to overcome this electrostatic repulsion. The aim of all surface immobilization methods is to secure the DNA with enough strength to the surface to facilitate consistent imaging at a good resolution but to leave the molecule enough flexibility to allow for any dynamics to be visualized—a “fixation-freedom” paradox. The principal methods used for DNA-mica surface immobilization include the use of divalent cations,^59^ silanization,^52,60^ and the use of cationic surfactants^61^ and polymers.^53^ Each method has its own advantages and drawbacks, which are reviewed here.

### Divalent ion mediated adsorption

One of the simplest methods to overcome the electrostatic repulsion between mica and DNA for AFM imaging is the divalent cation method, where cations such as magnesium (Mg^2+^) or nickel (Ni^2+^) are used to bridge the charged biomolecule and microscopy surface. A net attractive force is generated by associations between divalent counterions on the mica surface and negatively charged DNA, pulling DNA molecules to the surface.^62^ This phenomenon is especially effective if the surface charge density of the surface and polyelectrolyte are similar, as for DNA and mica. Early experiments used a modified transmission electron microscopy (TEM) protocol, during which freshly cleaved and sonicated mica was treated with magnesium ions to facilitate stronger DNA adsorption.^63^ Bustamante *et al.* (1992) used AFM to stably image a large three kilobase pair plasmid DNA in air using mica pretreated with magnesium ions.^55,64^ Subsequent work showed that pretreatment was not required as long as the ions were present in the buffer solution.^34,40^ The divalent cation protocols were extended to other ions including nickel and zinc, and a correlation between the hydrated atomic radii of the cation and DNA binding efficiency was found.^59^ DNA was observed to bind most tightly to mica when the ionic radii of the cations were 0.74 Å or less, e.g., with Ni^2+^ (0.69 Å).^59^ In this case, nickel ions form an adlayer on the cleaved mica surface.^65^ This is mediated by the exchange of native, highly mobile K^+^ ions on the mica surface with Ni^2+^ ions in solution, as shown by Time-of-Flight Secondary in Mass Ion Spectrometry (ToF-SIMS).^66^ The formation of a nickel adlayer facilitates strong binding of DNA to mica, with modulation of Ni^2+^ concentration shown to affect the translational freedom of the surface-bound DNA.^67^ The use of divalent ions for surface immobilization allows DNA molecules to deposit on the surface, equilibrating into their lowest energy 2D conformation.^68^

The majority of plasmid DNA samples imaged by AFM have been observed in the B-form DNA structure.^46,47^ However, divalent ion-treated mica can also affect the conformation of DNA on the surface, due to the intercalation of these ions into the DNA helix. This can result in shortening of the molecular contour length and partial B-form to A- and Z-form conformational transitions, as observed using AFM^50^ and confirmed by Tip-enhanced Raman Spectroscopy (TERS).^69^ High-resolution AFM allowed for further structural characterization of these unusual conformations in DNA, with this over-stretched plasmid DNA structure exhibiting a left-handed conformation with an elongated periodicity of 8.0 ± 0.5 nm. These molecules demonstrated an increased molecular contour length and were likely stabilized by the presence of nickel ions in the buffer solution.^50^

### Silanization

Another commonly used protocol for immobilizing DNA is silanization of the mica surface. First described by Lyubchenko and colleagues, mica is modified with 3-aminopropyltriethoxy silane (APTES) to obtain a positively charged AP-mica surface.^60^ APTES binds covalently to mica to create an AP-mica surface, which is positively charged through protonation of its amino groups. AP-mica facilitates stable binding of a range of double stranded DNA molecules, with the protocol optimized to reliably image larger DNA constructs such as the λ phage genome^70^ and shorter supercoiled DNA plasmids.^71^ In contrast to divalent cation-mediated immobilization, the surface adhesion in silanized surfaces is strong enough to result in kinetic trapping of DNA molecules on the surface. The conformations of the DNA molecules are therefore imaged as a 2D projection of the way the molecules are organized in three dimensions when in solution, without any equilibration.^72^ One benefit of this method is its use under a broad range of pH and buffer conditions; however, preparation of the silanized surface requires additional time and may also result in a rougher imaging surface.^73^ Additionally, aggregates of adsorbed APTES molecules can be commonly seen with APTES-mica due to the hydrolysis of APTES molecules and their rapid aggregation.^71,74,75^

In another silanization approach, aminopropyl silatrane (APS) uses silatranes instead of silanes as a means to mitigate some of the drawbacks associated with the fast hydrolysis of APTES.^52,76^ Similar to APTES, APS reacts with the hydroxyl groups on the surface of the cleaved mica, leading to the formation of APS-mica. As a result of their similar surface chemistry, reliable imaging of DNA molecules has been achieved with both APTES^77^ and APS.^78,79^

### Other immobilization strategies

A range of other surface modifications have also been used to immobilize DNA for imaging by AFM. These include the use of cationic surfactant bilayers to immobilize and image DNA plasmids in aqueous solution.^61^ Dense packing of the DNA on bilayers provided lateral stabilization of DNA, facilitating measurement of the periodicity of the packed DNA molecules as 3.4 ± 0.4 nm, consistent with the B-form structure. Other methods involve the use of gold surfaces where DNA immobilization is facilitated through thiol modification^80^ and non-treated glass, which was shown to bind chromatin.^81^ Cationic polymers such as poly-L-ornithine^82^ and poly-L-lysine (PLL)^45,83^ facilitate DNA adsorption for AFM imaging at high resolution in a range of buffer conditions, including those permissive to observation of DNA-protein binding events. Recent work has demonstrated the use of hydrophilic diblock copolymers of polyethylene glycol (PEG) and PLL (PLL-*b*-PEG) to achieve selective adsorption of DNA-protein complexes.^53^ At physiologically relevant protein concentrations, the inclusion of PEG minimizes the deposition of un-bound protein to the underlying substrate, which would otherwise conceal the visualization of DNA-protein complexes.

## AFM OPERATIONAL MODES FOR DNA IMAGING

Having optimized sample preparation for a given sample, a second variable, the operation mode of the AFM can significantly affect imaging resolution and reproducibility. The AFM can be operated in a variety of imaging modes, each with its own advantages and disadvantages. There are dozens of AFM modes with additional sub-categories, each with facets specialized to certain applications or fields of study.^84,85^ In general, AFM modes can be grouped as either dynamic or static. Static modes, for example contact mode, image by raster scanning the cantilever across the sample surface with its tip in constant contact. The lateral action of the tip in contact mode can be destructive, so this mode is most appropriate for mapping “solid” samples, such as nanomaterials.^86^ For “soft” samples, such as biomolecules and DNA, contact mode offers high scan speed (capable of real-time imaging). However, the samples must be fixed or arranged in conformations which resist lateral forces.^61^ Dynamic modes, such as tapping mode-based AFM techniques, reduce the lateral forces applied during scanning due to their intermittent tip-sample contact, which can be more appropriate for imaging of biomolecular samples.^87^

**FIG. 2.**
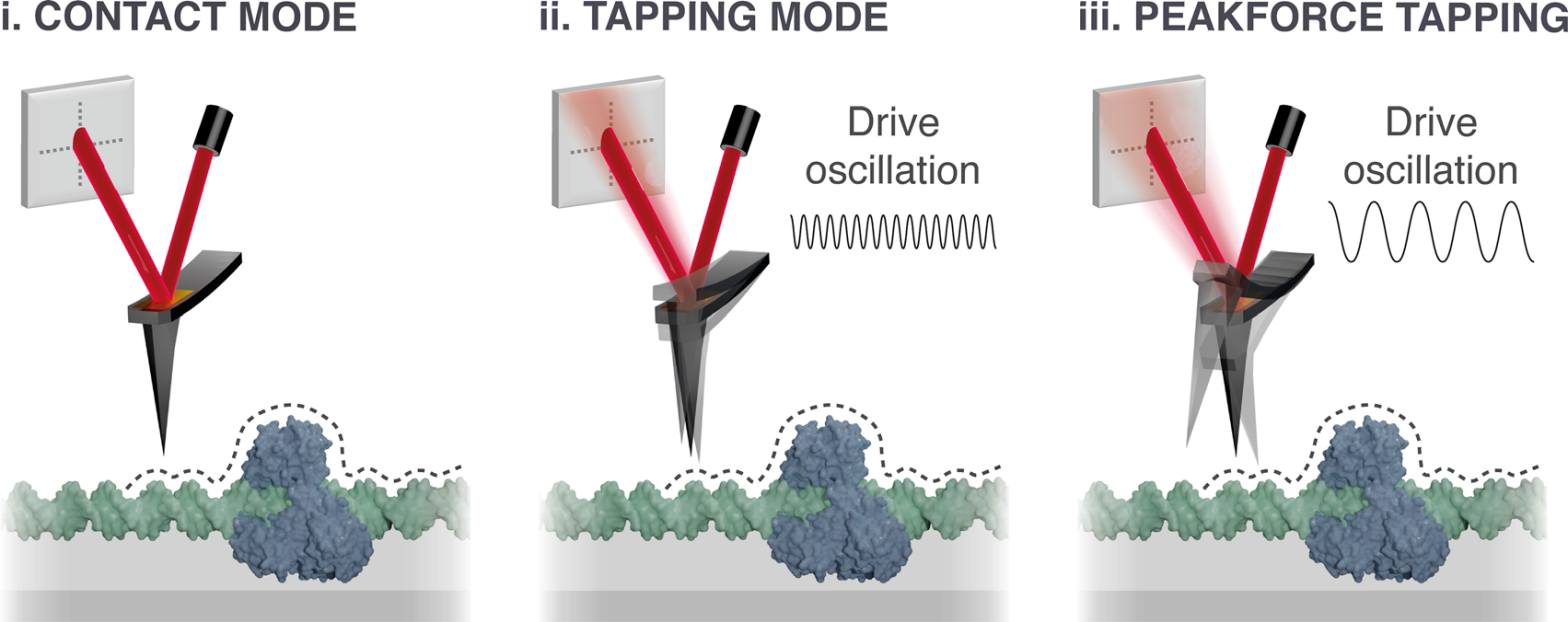
Schematic showing three key AFM imaging modes. For each mode, a cantilever-tip is raster scanned across a sample (dashed line). Surface features induce a change in the bending of the cantilever and therefore deflection of the incident laser, which is monitored by a quadrant photodiode. These changes are fed into a feedback loop to control tip-sample separation and provide a topographical map of the surface. In contact mode (i), the tip scans laterally without interrupting tip-sample contact, resulting in increased lateral forces. In dynamic modes such as tapping mode (ii) and PeakForce tapping mode (iii), the cantilever is driven to oscillate sinusoidally, resulting in intermittent contact with the surface and reduced lateral forces. In tapping mode, the cantilever is driven and oscillated close to its resonant frequency through a small amplitude of oscillation. In PeakForce tapping mode, the cantilever is driven at frequencies much lower than that of its resonant frequency through a larger amplitude of oscillation.

In most implementations of AFM imaging, a sharp tip (end radius in the nanometer size range) on the underside of a reflective cantilever is raster-scanned across a sample, building up a picture of the surface topography line-by-line (Fig. 2). As the tip follows the contours of the sample, the soft, flexible cantilever to which it is attached bends. The bending of the cantilever is tracked by the reflection of a laser source from the back side of the cantilever (usually plated with highly reflective materials such as gold) onto a four-quadrant photodiode (Fig. 2). The incident laser light reflected onto each photodiode quadrant is converted into a Voltage, which is tracked by the microscope controller.^88^ These voltages are fed into the feedback loop, the function of which is to keep the force being applied to the sample constant. The exact process by which this occurs depends on the mode of AFM being used. Here, common modes such as contact mode, tapping mode, and PeakForce tapping mode will be explored with a focus on their development in relation to DNA imaging.

### Contact mode

Contact mode was the first mode of AFM developed and within a year of its inception had been used to image the surface of graphite at atomic details.^22^ In contact mode, the sharp tip on the underside of a reflective cantilever is scanned across the sample line by line in an x-y raster scanning motion (Fig. 2-i). The contours of the sample are readout through the bending of the cantilever, as measured by the deflection of a laser source reflected from the back side of the cantilever. This bending is converted to a change in voltage by a photodetector and used as input to a piezoelectric motor, which adjusts the height of the cantilever or sample in the z direction during raster scanning. This maintains a constant interaction force between the cantilever-tip and sample. The laser deflection signal and z-axis adjustments are then used to build images of the surface topography.^88^ Cantilevers with low spring constants of <0.1 N m^−1^ are used for contact mode imaging of biological samples, with typical applied forces in the range of 100–500 pN, at scan rates around one frame per minute.^89^ However, the constant application of lateral forces during raster scanning can result in irreversible damage to soft biological samples; hence careful iteration of parameters is required.^90^ Due to these higher lateral forces, contact mode can be less effective for achieving high-resolution imaging if samples are not laterally stabilized, e.g., through incorporation in a 2D lattice.^91^ Lateral stabilization was used in an early example of contact mode for high-resolution imaging of DNA by adsorbing DNA at very high concentration on cationic surfactant bilayers.^61^ The closely packed nature of the DNA molecules allowed the helical pitch of DNA with a periodicity of 3.4 ± 0.4 nm to be resolved along the molecule, consistent with the hydrated B-form DNA structure.^61^ For imaging of less densely packed DNA, for example, isolated plasmids, the lateral forces of contact mode proved too high for many applications. The development of other modes such as tapping mode with lower lateral forces was therefore urgently needed.

### Tapping mode

Tapping mode (also known as intermittent contact mode or amplitude modulation AFM) is a dynamic AFM mode in which the sharp tip “taps” the sample intermittently (Fig. 2-ii).^92^ The advent of tapping mode greatly improved AFM imaging of DNA due to the reduction in lateral forces applied to the sample during imaging.^93^ The tapping motion is achieved by oscillating the cantilever above the sample in the z direction, at a frequency *f* close to the natural resonance of the cantilever, *f_0_*. Whilst the tip oscillates in z, the cantilever raster scans the surface in the x,y direction to build up a picture of the sample surface. The tip-sample separation is tracked indirectly by changes in the oscillation amplitude of the tip, which will decrease as the tip-sample separation decreases. The size of the cantilever oscillation is measured as the root mean squared amplitude of the deflected laser movement on the detector. This value is fed into the feedback loop to maintain a constant amplitude of oscillation of the cantilever by adjusting the position of the cantilever with respect to the sample (or vice versa), maintaining a stable tip-sample interaction force. The oscillation of the tip reduces the lateral frictional effects of raster scanning as the cantilever spends most of its travel time out of contact with the sample. Tapping mode is therefore particularly useful for imaging molecules loosely bound to a substrate with minimal movement. Tapping mode imaging of plasmid DNA in fluid was first carried out by Hansma *et al.*, who obtained comparatively high resolution images of DNA in water.^94^ This was demonstrated by the measured widths of DNA plasmids, which were observed to be around 5 nm, compared with 10 s of nanometers when imaged using contact mode in fluid.^70^ Another advantage of tapping mode is that it allows for loosely bound DNA to be imaged; thus the dynamics of DNA motion and degradation can be imaged in real-time.^35^

Although tapping mode reduces the lateral shear forces between the sample and tip compared to contact AFM modes, deformation by the oscillating tip is still common.^95^ Furthermore, in fluid, resolution may be affected due to the effects of fluid damping,^96^ which reduces the sensitivity of the method to changes in amplitude. Another drawback for tapping mode AFM imaging in fluid is the convolution of the cantilever resonance with the mechanical resonances of the fluid cell. This arises as the cantilever is driven by a piezo actuator which drives the entire fluid cell, or cantilever holder. This excites a variety of mechanical resonances in the fluid cell as well as the cantilever itself, resulting in an excitation spectrum that is commonly denoted as a “forest of peaks.”^96^ This forest of peaks can vary over time and as the fluid volume changes within the fluid cell. This can result in large changes to the amplitude of the cantilever resonance peak as the forest of peaks moves.^96^ Changes to the free amplitude of oscillation of the cantilever will result in changes in the applied force during imaging. This is a major drawback for the imaging of biomolecules in fluid using tapping mode, as although the lateral forces are reduced, the applied force is ill-defined and can vary substantially.^97–99^ The large changes in applied force can result in a loss of resolution and damage to the sample or to the tip. Given correct optimization of key parameters, however, tapping mode can provide high resolution imaging of biological molecules in fluid. This has been demonstrated for both DNA^47^ and double stranded RNA (dsRNA) molecules with sub-molecular resolution, resolving the helical pitch at 3.1 ± 0.3 nm consistent with A-form dsRNA.^48^

### Frequency modulated AFM (FM-AFM)

The use of frequency modulation AFM (FM-AFM) as a tool for high-resolution imaging substantially increased the level of detail observed in AFM images of DNA molecules in solution.^100^ In FM-AFM, a cantilever is oscillated at its resonance frequency, and the actuation frequency is continuously adjusted to track the resonance frequency of the cantilever.^101^ The tip-sample interaction is monitored directly via a shift in the resonance frequency of the cantilever, instead of via a change in amplitude as in tapping mode. In fluid, FM-AFM is performed in the repulsive force regime, such that the frequency of the cantilever increases as the tip-sample separation decreases.^102^ This allows small changes in tip-sample variation to be monitored as a comparatively large shift in the resonance frequency, which allows for greater force control and therefore measurements with sub-molecular resolution.^103^ Most FM-AFM systems are designed to work with small cantilevers, of length <10 *μ*m with resonant frequencies in the MHz range. These cantilevers have been used to image DNA plasmids at a rate of 0.2 frames per second, determining helical pitch of the double helix to be 3.4 nm, in good agreement with x-ray crystallography.^104^ Further work using FM-AFM was achievable high-resolution of the DNA double-helix, allowing for the major and minor grooves to be resolved,^50^ as well as periodic corrugations corresponding to individual phosphate groups in the DNA backbones.^46^ This allowed for variation in the handedness of DNA molecules to be observed for individual molecules.^50^ These studies demonstrated the power of AFM to provide insight into variations in the DNA structure at the sub-molecular scale.

### PeakForce tapping

PeakForce tapping (PFT), a rapid force-distance imaging mode, is a relatively new dynamic imaging mode.^105,106^ In PFT, the cantilever is driven in a sinusoidal motion at a frequency much lower than its resonant frequency *f_0_* (Fig. 2-iii). A force curve is recorded at every oscillation and the tip-sample interaction controlled by a feedback loop, setting the maximum applied force or “peak force” between the tip and the sample for each curve. The peak force from each curve is then used as an input to modulate the z-piezo position and maintain a constant tip-sample interaction force, reducing the potential for tip-sample interaction deformation or damage.^107^ The initial implementation of force-distance measurements in AFM was exceptionally slow, with the generation of a single force curve taking between 0.1 and 10 s, equaling potentially hours to image a tiny 32 × 32 pixel area.^108,109^ Scan rate improvements in PFT with respect to traditional force-distance imaging have enabled high-resolution scanning (e.g., 512 px^2^) in under 10 min.^49^ The current scan rates in PFT permit either extremely high-resolution scans of single molecules (low scan area, many tip-sample interactions) or a high volume of scans at low resolution (large scan area, few tip-sample interactions). Although PFT is still substantially slower than tapping mode, work by Nievergelt *et al.* has reduced this disparity through the implementation of photothermal off resonance tapping (PORT) in which the cantilever is directly actuated to achieve a two orders of magnitude increase in measurement frequency.^110^

One major advantage of PFT is that it refers the measured peak deflection (and thus force) to the baseline cantilever deflection away from the surface and thus is able to compensate for drift. This allows for imaging of soft biomolecules, such as DNA, over extended periods of time with minimal tip damage.^49^ PFT allows for stable imaging of DNA in fluid at a resolution comparable to that obtained in tapping mode, but with the added advantage of more stable and sustained imaging. PFT has been used to observe variations in a double helical structure along a single plasmid of DNA visible as double-banded corrugation along the molecule.^47^ Here, it was demonstrated that imaging at high (∼200 pN) forces results in a loss of sub-molecular resolution and excessive deformation of the sample. However, when imaging at low peak forces (∼40 pN), the plasmids were shown to barely deform, with height measurements in good agreement with the crystal structures of B-form DNA (1.9 ± 0.2 nm).^111^

## OTHER FACTORS AFFECTING AFM IMAGE ACQUISITION AND ANALYSIS

### Photothermal actuation of the AFM cantilever

Due to their bimetallic nature and propensity to bend,^112^ AFM cantilevers can also be actuated by local laser heating, known as photothermal actuation,^113,114^ to improve image stability. This improvement is due to the elimination of the so-called forest of peaks in the resonance spectrum, caused by excitation of the support chip or other spurious resonances in the fluid cell. In photothermal actuation, the cantilever is brought to resonance by focusing a second laser, known as the actuation laser on the back surface of the cantilever and modulating this at the resonance frequency of the cantilever. This method is particularly useful when imaging in fluid as photothermal actuation does not require any additional electrical connections or corrosive coatings to the cantilever. The laser modulation also allows for the use of both standard and small cantilevers with ∼ MHz resonance frequencies. This is particularly important for further miniaturization of cantilevers and for the corresponding increase in resonance frequencies^50,103,115–117^ as problems related to spurious mechanical resonances are aggravated when cantilever resonance covers a broader frequency spectrum. The amplitudes achieved by photothermal actuation are, however, rather small.^113,118,119^ Several methods have been used in order to extend this amplitude range including exploiting the trapezoidal form of the cantilever cross section,^120^ blackening of cantilevers by a sputtered gold palladium coating to enhance light adsorption,^113^ and coating of cantilevers with an amorphous carbon layer to increase heat absorption.^121^ Photothermal actuation has been used in tapping mode to visualize DNA,^122^ the self-assembly of proteins^98^ and live cells,^110^ in FM-AFM to image DNA,^50^ and PFT to study the kinetics of the membrane attack complex pore assembly.^123^

### The role of the AFM tip in image resolution

The resolution of AFM is also limited by the cantilever-tip and the forces it enacts upon the sample. These have important implications for the interpretation of structures observed in AFM images^124^ and particularly the double helical structure of DNA.^46,47^ To illustrate these boundaries of resolution, we can consider a B-form dsDNA helix with a diameter of ∼2 nm. For comparison, the sharpest commercially available AFM tips have nominal radii of ∼1 nm. If we assume a common imaging situation where there is a scan resolution of 1 nm/px, then as the tip scans the molecule, its width means that it feels the surface of the DNA with the side of the tip, but registers the position as if it was the center. This means that there is a correct measurement only at the very center of the molecule, but as the tip begins its downward arc at the side of the helix, there is tip-convolution. This is where the effective radius of the molecule of interest is increased (e.g*.,* a DNA molecule which appears 2.5–10 nm in diameter) because the measurement is convoluted with the radius of the cantilever-tip.^47,50^ This does not affect contour length measurements of a DNA molecule, but tip-convolution has implications for the interpretation of the helical repeats, as they can be exaggerated non-uniformly by the widening effect.^46,47^ This issue would be similarly encountered for other molecules of similar size to the AFM tip, e.g., protein complexes.

The maximum topographical resolution achievable on soft flexible biomolecules, e.g., DNA in AFM is a fine balancing act between the tip-sample interaction forces required to recognize features, and the maximum forces the sample can accommodate before it distorts or deteriorates. If a tip-sample interaction force is too great, then imaging quality can deteriorate due to compression of the subject. For example, the topography of a DNA molecule was observed to compress by almost twofold between sequential scans at 39 pN and 193 pN.^47^ Additionally, even in dynamic AFM modes, if the interaction forces are too high, the subject molecules can be “kicked around” by the tip-sample interaction and raster scanning motion, leading to poor scan quality.

### Development of AFM image analysis tools

Despite many hardware developments in high-resolution bio-AFM, one of the biggest challenges remains in the analysis of increasing volumes and complexity of data produced. Traditionally, the majority of AFM analysis has been carried out by hand, relying on a highly trained and experienced researcher. When coupled with data acquisition that is highly dependent on the expertise of the operator, this has limited the adoption of AFM as a tool that can solve problems inaccessible to cryo-electron microscopy (Cryo-EM), x-ray crystallography, or NMR. In contrast to other single molecule techniques such as Cryo-EM which has recently seen a “resolution revolution”^125^ in terms of investment in image and analytical processing infrastructure, the use of AFM as a quantitative imaging technique has been limited. Recent efforts have attempted to address this need through the development of novel automated analysis methods for various interests including DNA-bound protein conformations,^126,127^ nucleosome conformations,^128,129^ global DNA curvature,^130^ and DNA bend angles within DNA-protein complexes such as that of DNA and glycosylases.^131^ These present a method of high-throughput analysis across large amounts of data with minimal selection bias by being investigator-independent. Another example includes the development of TopoStats, an open-source Python utility that allows for single molecule identification and tracing of complex heterogeneous DNA populations as well as biomimetic pores.^132^ The creation of these utilities should not only facilitate and accelerate high-throughput AFM image processing and analysis but should foster community-led development toward more complex analysis.

## DETERMINING THE STRUCTURAL AND MECHANICAL PROPERTIES OF DNA USING AFM

Since the discovery of the double helical structure of DNA, there has been a drive to understand the complex mechanical and structural properties of DNA and uncover how topological strain and compaction within the cell affect its biological function. DNA topology is tightly regulated within cells and has been reviewed in detail many times in recent years.^133–135^ Traditionally, imaging of DNA has been carried out using electron microscopy techniques to investigate various parameters including measurements of twist and writhe,^6,136,137^ supercoiled linkage,^138,139^ the formation of bends,^7,8^ cooperative kinks,^140^ and knotting/catenation.^141,142^ However, most studies of DNA by electron microscopy are of limited structural resolution as uncoated DNA lacks contrast against the sample grid (in the case of planar TEM). Meanwhile, the conformational diversity of DNA prevents ensemble averaging by Cryo-EM outside of very short molecules.^8^

### AFM imaging of the DNA structure

**FIG. 3.**
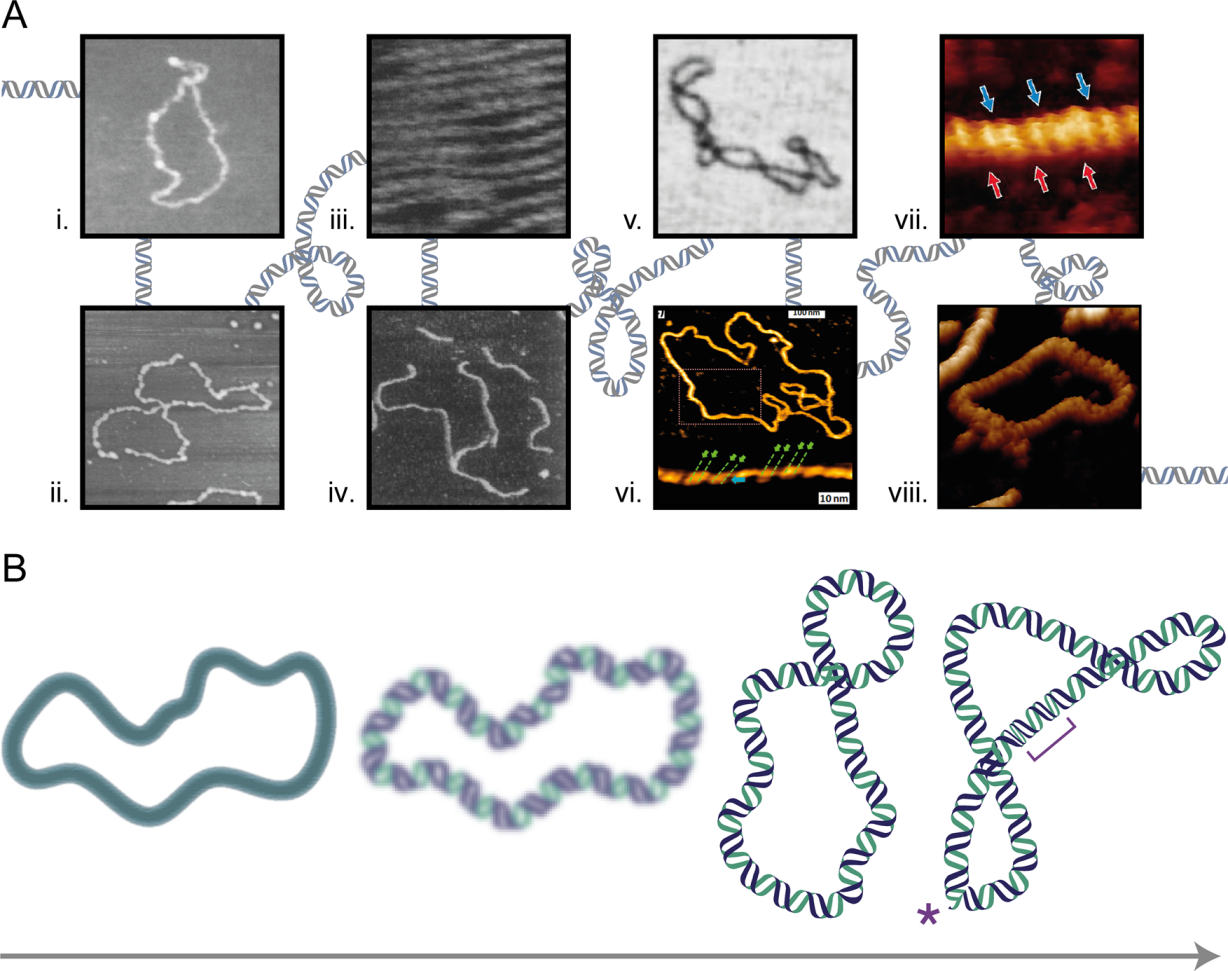
Timeline showing the progress of DNA imaging by AFM, from early images of DNA in air to high-resolution mapping in fluid. (a) DNA plasmids imaged in (i) air^55^ [Reprinted with permission from Bustamante *et al.*, “Circular DNA molecules imaged in air by scanning force microscopy,” Biochemistry **31**, 22–26 (1992). Copyright 1992 American Chemical Society]. (ii) aqueous solution^143^ [Reprinted with permission from Hansma *et al.*, “Atomic force microscopy of DNA in aqueous solutions,” Nucl. Acids Res. **21**(3), 505–512 (1993). Copyright 1993 Oxford University Press]. (iii) Immobilized on a cationic supported surfactant bilayer^61^ [Reprinted with permission from Mou *et al.*, “High-resolution atomic-force microscopy of DNA: the pitch of the double helix,” FEBS Lett. **371**(3), 279–282 (1995). Copyright 1995 John Wiley and Sons]. (iv) Immobilized using Ni^2+^ cations^59^ [Reprinted with permission from H. G. Hansma and D. E. Laney, “DNA binding to mica correlates with cationic radius: Assay by atomic force microscopy,” Biophys. J. **70**(4), 1933–1939 (1996). Copyright 1996 Elsevier]. (v) Immobilized on APTES-functionalized mica^78^ [Reprinted with permission from Y. L. Lyubchenko, “DNA structure and dynamics: An atomic force microscopy study,” Cell Biochem. Biophys. **41**, 75–98 (2004). Copyright 2004 Springer Nature]. High-resolution AFM images of the DNA helical structure, able to discern; (vi) the handedness of individual DNA molecules^50^ [Reprinted with permission from Leung *et al.*, “Atomic force microscopy with nanoscale cantilevers resolves different structural conformations of the DNA double helix,” Nano Lett. **12**, 3846–3850 (2012). Copyright 2012 American Chemical Society]; (vii) individual phosphate groups in the DNA backbone.^46^ [Reprinted with permission from Ido *et al.*, “Beyond the helix pitch: Direct visualization of native DNA in aqueous solution,” ACS Nano, **2**, 1817–1822 (2013). Copyright 2013 American Chemical Society] (viii) and kinks and defects^147^ [Reprinted with permission from Pyne *et al.,* “Base-pair resolution analysis of the effect of supercoiling on DNA flexibility and major groove recognition by triplex-forming oligonucleotides,” Nat. Commun. **12**, 1053 (2021). Copyright 2021 Authors, licensed under a Creative Commons Attribution (CC BY) license]. (b) Schematic showing progress in AFM imaging of DNA, from low resolution imaging of molecular conformation, to double the helical structure including changes in intramolecular groove size (bracket) and defects (asterisk).

Early AFM studies of DNA had poor structural resolution due to movement and distortion of the DNA molecules^31,32,143^ [Figs. 3(a-i) and 3(a-ii)]. Figure 3 shows how the rapid development of improved sample deposition methods and tapping mode AFM (discussed above) quickly allowed AFM to reveal the structure of DNA. AFM imaging has been used to determine a range of structural parameters including handedness, major/minor groove angles, and periodicity [Fig. 3(a-iii)] which were all found to be in agreement with the structural characteristics of DNA proposed by x-ray crystallography studies.^61,144–146^ Developments in DNA immobilization methods improved both the resolution and reproducibility of AFM images^59^ [Fig. 3(a-iv)], through which different DNA conformations were also studied^78^ [Fig. 3(a-v)]. More recent advances in AFM, such as PFT mode and cantilever design refinements, have allowed high-resolution periodicity measurements of the major and minor grooves of a single DNA molecule^50^ [Fig. 3(a-vi)] and visualization of individual phosphates in the backbone of DNA^46^ [Fig. 3(a-vii)]. This has been followed by reproducible visualization of the secondary structure of uncoated DNA under aqueous conditions in which intramolecular variations of groove depths were observed, along with direct measurements of twist^147^ [Fig. 3(a-viii)]. AFM has also been used to observe uncommon DNA configurations such as Z-DNA,^148^ triplex-DNA,^149–152^ and G-quadruplex DNA^153–156^.

### AFM studies of the effect of supercoiling on the DNA structure and conformation

In a covalently closed circular DNA molecule, the relationship between the two helically inter-wrapped single DNA strands is fixed, unless one or both DNA strands is cleaved. This topological relationship is known as DNA linkage and means that two molecules of identical base-pair number can have different topological configurations (topoisomers) based on the number of helical turns amongst the two single strands (twist). Deviations from the “ideal” ∼10.5 bp per helical turn of B-DNA are energetically compensated by supercoiling, where the dsDNA coils (writhes) around its own axis. Most cellular DNA is maintained in an under-wound state (>10.5 bp/turn), as the corresponding negative supercoiling is advantageous from the perspectives of genome compactivity and a reduced energetic cost to proteins for DNA melting.^157^ The effect of supercoiling upon the conformations of DNA adopted on the mica surface has been revisited several times.^158–161^ The recent study by Bettotti *et al.* demonstrated that positively and negatively supercoiled DNA behave differently upon mica in an adsorption-dependent manner. Highly negatively supercoiled DNA demonstrated “open” non-writhed configurations when imaged on mica functionalized using the divalent cation method (Mg^2+^), while the same DNA sample was highly plectonemically writhed if deposited using APTMS-functionalized mica. Meanwhile, extensively positively supercoiled DNA was highly writhed by both deposition methods, suggesting a specific interaction between negatively supercoiled DNA and the cation-deposition method.^158^ Many studies of DNA damage by radiation have used AFM to quantify the proportions of supercoiled, nicked, or linear DNA remaining in a sample post-exposure and made comparisons between these data with other techniques such as gel-electrophoresis.^162–167^

### AFM studies of minicircle DNA

Small covalently closed circular DNA minicircles of length <500 bp have been demonstrated to be useful tools for investigations of changes in DNA conformation and structure in response to biologically relevant phenomena, e.g., modified levels of supercoiled linkage (ΔLk). The limited length of the DNA circles is ideally suited to structural evaluation by AFM at high-resolution, as their small size, only 2–3 persistence lengths, results in a conformational landscape with minimal complexities in the form of trivial crossings. AFM has facilitated determination and analysis of the entire conformational landscape of these structures by imaging large populations of individual molecules.^147,156,168^ Fogg *et al.* used AFM (tapping mode AFM in air, dehydrated samples) to study negative supercoiling in 339 bp DNA minicircles. This study demonstrated that increasing levels of negative supercoiling gave rise to diverse conformational heterogeneity, as observed by AFM micrographs of “open” circular minicircles at the lower levels of ΔLk, contrasted with ‘rod-like’ tightly compacted minicircles at high levels of ΔLk.^168^ This minicircle conformational diversity phenomenon was later re-investigated using single-particle Cryo-EM, of both positively supercoiled and negatively supercoiled 339 bp minicircles, where it was discovered that highly writhed conformations observed at the highest levels of ΔLk were facilitated by local disruptions (e.g., kinks and defects) in the DNA helix.^9^ High-resolution AFM was used to determine the twist of these molecules and demonstrate that these small compact, defect containing structures exist in the canonical B-form.^147^ Beyond twist determination, AFM was able to determine the exact location of these defects and correlate their formation to conformational changes in DNA minicircles with controlled levels of superhelical stress. This demonstrated the complementarity of AFM with other biochemical and structural techniques to determine the structure of complex DNA under bending and superhelical stress.

### AFM studies of topologically complex DNA

A covalently closed circular DNA molecule which is self-entangled is a DNA knot. If two (or more) closed DNA circles are interlinked, they form a DNA catenane. Like topoisomers, DNA knots and catenanes are topological configurations, which cannot be interconverted without double-strand cleavage, and they can be classified by both the minimal number of crossings in their structure, and the chirality of those crossings. To date, studies of plasmid-scale DNA knotting and catenation with AFM have been rare. The first observation of catenated plasmid DNA was performed by Yamaguchi *et al.*, which presented a single image of a DNA catenane captured at low resolution.^169^ Subsequently, the work of Harmon *et al.* observed heterogeneous mixtures of pUC19 plasmid (2686 bp) catenanes, where some were multiply interlinked. Interestingly, the study also utilized RecA-coating of the catenated DNA to attempt to improve the resolution of the interlinked DNA crossover, although the precise topological chirality of the catenanes was not the focus of the investigation.^170^

The highly catenated mitochondrial DNA of trypanosomatid species (kDNA) has also been observed by AFM, where upon treatment with Human Topoisomerase II, the kDNA network was observed by AFM to decatenate into ∼2.5 kb circles with sequentially fewer interlinks, with sufficient scan resolution to discern the directionality of the DNA crossovers.^171^ Knotted DNA has been examined by AFM on fewer occasions than catenanes, and never yet with sufficient resolution to discern the complex topological configurations of the molecules.^172–174^ These studies examined the knotted 11.6 kb genomic DNA of bacteriophage P4; however, the highly knotted mixtures of phage-DNA were topologically unclassifiable due to low imaging resolution. López *et al.* attempted to determine whether the partially replicated plasmid DNA from Topoisomerase IV-deficient *E. coli* contained interchromatid knotting. Interestingly, RecA-coating was used during this study to exaggerate the thickness of the DNA strand crossovers and ease in assigning under/over-crossing strand chirality, just as the original electron microscopy studies of knotted DNA had done so.^175^ Determining whether juxtaposed DNA strands are crossing over or under each other is a challenging proposition, which has not yet been fully explored by AFM but is necessary to differentiate the absolute configurations of knotted or catenated DNA molecules. The aforementioned recent advances in DNA-AFM resolution may yield sufficient resolution to differentiate DNA crossover directionality without coating by RecA; however, this has yet to be demonstrated. Beyond studies of DNA alone, this shows the promise of AFM as a technique to probe the effect of DNA topology on DNA-protein interactions and gauge the influence of structural specificity on key biological mechanisms.

## AFM STUDIES OF DNA–PROTEIN INTERACTIONS

**FIG. 4.**
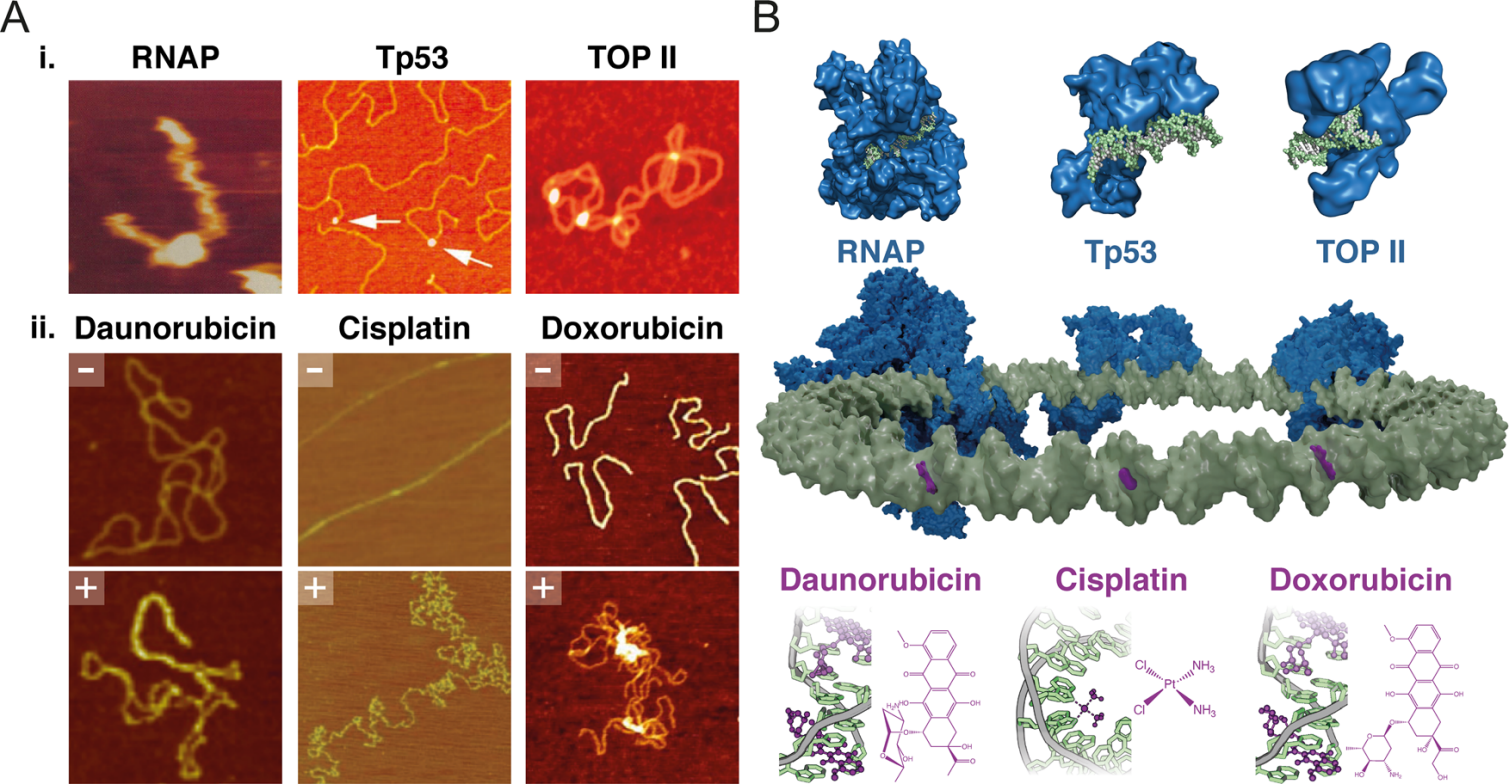
(a) Corresponding AFM images showing (i) the interaction of RNAP^176^ [Reprinted with permission from Guthold *et al.,* “Following the assembly of RNA polymerase-DNA complexes in aqueous solutions with the scanning force microscope,” Proc. Natl. Acad. Sci. U. S. A. **91**, 12927–12931 (1994). Copyright 1994 National Academy of Sciences, U.S.A.], Tp53^180^ [Reprinted with permission from Jiao *et al.*, “Dynamic interactions of p53 with DNA in solution by time-lapse atomic force microscopy,” J. Mol. Biol. **314**(2), 233–243 (2001). Copyright 2001 Elsevier] and TOPII^174^ [Republished with permission from Alonso-Sarduy *et al.*, “Human topoisomerase II-DNA interaction study by using atomic force microscopy,” FEBS Lett. **585**(19), 3139–3145 (2011). Copyright 2011 Elsevier and Clearance Center, Inc.] with DNA and (ii) DNA architecture in the absence (-) and presence (+) of small molecule therapeutics^181–183^ [Reprinted with permission from Alonso-Sarduy *et al.*, “Time-lapse AFM imaging of DNA conformational changes induced by daunorubicin,” Nano Lett. **13**, 5679–5684 (2013). Copyright 2013 American Chemical Society]. [Reprinted with permission from Hou *et al.* “Cisplatin induces loop structures and condensation of single DNA molecules,” Nucl. Acids Res. **37**, 1400–1410 (2009). Copyright 2009 Authors, licensed under a Creative Commons Attribution (CC-BY-NC) license]. [Reprinted with permission from Cassina *et al.,* “Atomic force microscopy study of DNA conformation in the presence of drugs,” Eur. Biophys. J. **40**, 59–68 (2011). Copyright 2011 Springer Nature]. (b) Schematic of DNA with bound proteins (blue) and small molecule therapeutics (purple). Protein crystal structures are shown above (PBD ID's: 5FJ8, 1TUP, and 4FM9) and small molecule therapeutics below (PDB ID's: 1DA0, 2NQ0, and 1D12).

DNA-protein interactions are at the heart of cell viability, and many are implicated in cancer or are direct targets for anti-cancer therapeutics. In this section, we will cover how innovations in dynamic AFM imaging modes, improvements in force control and resolution, and the development of softer cantilevers have increased the use of AFM to understand DNA-protein interactions relevant to cancer progression or inhibition. Figure 4 shows some examples of these interactions, with AFM images shown in Fig. 4(a) and corresponding crystal structures in Fig. 4(b). One of the earliest examples of AFM being used to evaluate DNA-protein interactions was in 1994 where the innovation of tapping mode^94^ allowed AFM to be used to observe the motion and enzymatic degradation of DNA.^35^ The motion of small 324 bp pieces of DNA was observed by animating consecutive image scans and the amount of degradation inferred from the accumulation of debris in time-lapsed consecutive images. The authors of this work concluded that in order to capture enzymatic activity via dynamic AFM imaging, the temporal resolution of AFM must be improved through developments that enable faster scan rates. Nonetheless, this was a successful early example in which the coupling of technological and chemical advancements, in this case the binding strength of DNA to the surface, provided insight into a key biological interaction.

Early AFM experiments characterized the interaction of the essential enzyme RNA polymerase (RNAP), which initiates transcription. Guthold *et al.* observed nonspecific complexing of RNAP with 1258 bp DNA fragments [Fig. 4(a-i)].^176^ Nonspecific binding of the RNA polymerase to the DNA was observed, without diffusion of the polymerase along the DNA molecule, possibly due to the stable attachment between DNA and the mica surface. This early AFM imaging was particularly affected by hydrodynamic drag forces and thermal changes upon introduction of liquid into the cell for the first time, requiring long waiting times for the AFM to reach a mechanical and thermal equilibrium. However, this work demonstrated the capability of AFM to observe and characterize macromolecular assembly; the precursor to dynamic enzymatic processes. The second study utilized 1047 bp DNA molecules and aimed to investigate the dynamic interactions between *E. coli* RNA polymerase and DNA, using tapping mode.^177^ Aside from observing assembly, processive movement in the form of RNAP diffusion along nonspecific DNA was imaged, whereby the RNAP appeared to slide back and forth along the DNA before it was released. The dynamic movement of other proteins have been visualized by high-speed AFM, such as RAD54, which was shown to diffuse and hop along DNA molecules.^178^ High speed AFM has also been used to map specific locations of DNA-protein interactions along linear DNA molecules where bound proteins are correlated with target sequence locations.^179^ This allows for AFM to be used as a complementary sequencing tool.

AFM has been used to investigate the dynamic interactions of transcription factors including p53, a key tumor suppressor protein, with DNA. p53—DNA complexes were imaged in air, and p53 interactions with DNA were imaged in liquid.^180^ p53 was found to bind nonspecifically to plasmid DNA [Fig. 4(a-i)]; a two-step binding mechanism was resolved involving nonspecific binding of p53 to DNA followed by one-dimensional diffusion along the DNA molecule. However, p53 molecules were also seen to directly bind to one end of the DNA molecule, at the site of specific cloned binding sequences. Hence, some interactions between DNA and p53 were considered to be on the basis of partial specificity. Subsequent experiments were able to provide insight into the binding efficiency of p53 on DNA consensus sites and observe the formation of p53 dimers and tetramers.^184^ These studies demonstrate how AFM is able to observe multiple mechanisms of interaction through versatile design of DNA substrates and observation of multiple interactions.

AFM has also been used to determine the oligomeric state of DNA binding proteins. APOBEC3A (A3A) is a monomeric protein, one of seven human APOBEC3 DNA cytosine deaminases, and is known to have roles in foreign DNA degradation, inhibition of exogenous virus replication, and deamination. A3A was found to exist in a monomeric state in solution, irrespective of protein concentration even when complexed with ssDNA.^185^ A3A was found to bind both ssDNA and dsDNA, but with a much lower affinity to dsDNA (approximately 20% of binding events) using a hybrid DNA substrate: 69 nucleotide long ssDNA flanked by duplexes (hybrid gap-DNA). However, formation of complexes between A3A and hybrid DNA required a high molar ratio of A3A to DNA, possibly reflective of the transient interaction between the two. Prior to this study, limited information was available on the in-solution conformational state of A3A (monomeric or oligomeric). AFM facilitated single-molecule studies of protein conformations across a population showing A3A existing predominantly as a monomer, as compared to other techniques that rely on ensemble averaging that may favor a single conformational state.

A protein complex which plays an important role in DNA double-strand repair and genomic maintenance is the Mre11/Rad50/Nbs1 (MRN) complex. The MRN complex consists of two Mre11 exonucleases, two Rad50 ATPases, and a third nibrin (Nbs1) subunit in humans.^186^ High-speed AFM was used to determine the global architecture of these human, yeast, and bacterial complexes including the impact of the Nbs1 subunit on the human MRN complex.^187^ Tatebe and colleagues^187^ showed that the ring structure of Mre11/Rad50 repeats an open-close action at the head, but the hook between the Rad50 dimer remains closed. They demonstrated that the global architecture and conformational features of the Mre11/Rad50 complex were conserved.

The polycomb repressive complex 2 (PRC2) is a histone methyltransferase with critical roles in epigenetic gene silencing and heterochromatin formation.^188^ AFM was used to determine the sequence-specific binding of PCR2 with no spatial preference observed.^189^ This study used specific constructs containing a CpG island embedded in an otherwise low GC plasmid DNA construct. Unexpectedly, at high concentrations, PRC2 was found to compact the DNA via intramolecular loops involving PRC2 dimers or multimers. On binding, predominantly as a monomer, PRC2-induced bending of DNA was observed, with a threefold increase in the average value of the bend angle. Furthermore, by testing three different PRC2 composite complexes in the same study, it was found that only the full PRC2 complex demonstrated tight binding with DNA, in agreement with previous studies.^190^

Nucleosomes are the essential organizing subunit of the eukaryotic genome and consist of a histone octamer around which 50 nm of DNA (147 bp) is wrapped around 1.7 times. Nucleosomes are key in condensing DNA into the nucleus and also in DNA processing interactions. Establishing the dynamics of nucleosome assembly and disassembly is necessary to improve our understanding of the function of the genome. In a study by Katan *et al.* AFM imaging was used to show that nucleosomes disassemble spontaneously, on the order of approximately 1 s.^191^ However, the release of component units from the DNA was found to be at a slower rate, with nucleosome components remaining for tens to hundreds of seconds on the DNA. The study also observed the highly dynamic behavior of tetrasomes, which wrap ∼80 bp of DNA, showing a hopping and sliding translocation mechanism between stable positions along the DNA. Tetrasome disassembly was also accompanied by the formation of a DNA loop, showing key structural differences in the composition, conformation, and dynamics of nucleosome family members. High-speed AFM has also been used to investigate the conformational dynamics of Abo1: the fission yeast homolog of ATAD2—a histone chaperone implicated in nucleosome density regulation.^192^ Stochastic ring symmetry breaking was observed in real time by high-speed AFM, as individual blades of the ATAD2 hexameric ring being removed in the presence of ATP. This result correlated with Cryo-EM observations, providing insights into ATP-dependent histone deposition in nucleosome assembly and showing the power of AFM for dynamic structural characterization.

Topoisomerases are ubiquitous enzymes that modulate the topology of DNA. The dynamic interplay between topoisomerases and DNA has long been the subject of debate, with many topoisomerase structures yet to be crystallized. AFM imaging allowed for the first visualization of human topoisomerase II (TOP2), in physiological environments. Top2 was observed as homodimers with two distinguishable domains per monomer,^174^ [Fig. 4(a-i)]. Another mechanistic feature of type IIA topoisomerases, including TOP2, observed by AFM was the bend introduction in G-segment DNA^193^ as initially characterized by electron microscopy studies.^194^ The degree of bending was found to be less than that predicted by the EM bend model, thereby reemphasizing the need for multi-technique investigation. Beyond static studies of the topoisomerase structure, time-lapse AFM imaging was used to observe unknotting of knotted DNA in reaction with TOP2, demonstrated through an increase in radius of gyration of the DNA molecules (decreasing compaction) over time.^181^ This example highlights the power of AFM to detect local and global changes in individual DNA molecules on interacting with enzymes critical to the correct functioning of the genome.

## APPLICATIONS FOR AFM IMAGING OF DNA COMPLEXES IN CANCER RESEARCH

The interactions of DNA are not confined to enzymatic phenomena and also include a range of synthetic or modified ligands in the form of therapeutic agents, which interact with DNA directly or indirectly, to induce a specific pharmacologic effect. Many such small molecules are chemotherapeutics, which form one of the cornerstones of cancer therapy. However, cancer remains a leading cause of death, and with an annual predicted incidence of 27.5 × 10^6^ new cases by 2040,^195^ a strong onus remains on the need for improved therapeutics. The ability of AFM to probe DNA and enzyme structure with sub-molecular resolution on individual molecules in solution makes it an emergent tool for analyzing ligand-target interactions with scope to contribute toward optimization of current chemotherapeutics and the development of new drugs.

Intercalating agents have a wide variety of uses as chemotherapeutics. Anthracyclines such as doxorubicin (DOX) belong to this class and are widely used in solid and hematological malignancies.^196^ By intercalating between DNA base pairs, anthracyclines are thought to inhibit topoisomerase II activity during replication, causing subsequent arrest of the cell cycle and apoptosis.^197–199^ Cassina *et al.* investigated the interaction between intercalating agents DOX and ethidium bromide (EtBr) with plasmid DNA [Fig. 4(a-ii)].^183^ For both agents, the DNA appeared morphologically unmodified at lower concentrations of ligand, while the formation of plectonemic structures and aggregates were observed at higher concentrations. Of note, aggregate formation was seen at much higher concentrations of ligand for EtBr compared to DOX owing to the amino-sugar moiety in DOX increasing its affinity to DNA. This aggregation phenomenon has also been linked to DNA cleavage experiments, during which reduced DNA damage was observed at high concentrations of DOX.^200^ The aggregation prevents TOP2 access to DNA, leading to the formation of fewer cleavable complexes. These morphological insights provide a better understanding of the interplay between pharmacologic effect and structural dependence, an important consideration in therapeutic evaluation.

Daunorubicin (DAU) is another anthracycline known to intercalate between adjacent bases in double-stranded DNA. DAU induces local unwinding of the helix^201^ reducing the level of negative supercoiling in plasmids, and in some cases inducing positive supercoiling as shown by temperature gradient gel electrophoresis (TGGE)^202^ and AFM.^203^ In solution, negatively supercoiled DNA plasmids were shown to relax as DAU concentration increased, and at higher concentrations, positively supercoiled plectonemes were formed [Fig. 4(a-ii)].^181^ The liquid environment not only allowed changes to be monitored *in situ*, but also provided a basis for drug-DNA characterization at a higher spatial and temporal resolution in physiologically relevant conditions.

The “alkylating-like” platinum compounds including cisplatin are amongst the most common class of anti-cancer therapeutics. Cisplatin cross-links DNA in the major groove, forming adducts, culminating in the activation of apoptotic signaling.^204^ The major purine intrastrand cross-link is thought to bend the major groove of DNA and consequently widen the minor groove.^205,206^ Despite years of successful clinical use of cisplatin, a complete understanding of the structural changes it induces, such as local distortions and degrees of bending in DNA, are not fully understood, despite being considered the basis of the pharmacological activity of the drug.^207,208^ Bend induction and local flexibility at the site of binding have shown large variability when measured using NMR and x-ray crystallography.^209–212^ AFM provides a route to determine the origin of this large variability, through analysis of the entire conformational landscape of cisplatin-DNA interactions. One AFM study investigated the interaction between cisplatin and 300 bp DNA molecules that contained a single central cisplatin binding site.^208^ The induced bend angle was found to be 36°, with increased flexibility around the flanks of the bend. This is in good agreement with other AFM studies investigating this interaction where analysis was conducted using the worm-like chain (WLC) model to study a single cisplatin modification [Fig. 4(a-ii)].^182^ These studies demonstrate how AFM can provide a better understanding of DNA conformational properties, in the presence and absence of chemotherapeutics. These properties may affect not only the function of chemotherapeutics, but also protein recognition and binding that can lead to apoptosis and affect chemotherapeutic efficacy.

In the drive for the development of new chemotherapeutics, existing molecules, such as the polyamine analogue norspermidine (NSPD), are being explored. NSPD is thought to displace natural polyamines from their regulatory cellular functions.^213^ Studies of trinuclear norspermidine complexes with platinum or palladium were shown to have antitumor effects on breast cancer cell lines.^214^ Although the effect of the metal on cytotoxicity should also be considered as the palladium-NSPD complex was found to be more efficacious on particular cell lines than the platinum-NSPD complex. An AFM study aimed to quantify the morphological changes in DNA induced by polyamines, as these changes are thought to affect gene expression.^215^ Working on the principle that polyamines exert biphasic effects, enhancement and inhibition and that valence is a key consideration in DNA compaction, this study compared the effect of trivalent polyamines, spermidine (SPD), and NSPD, on the DNA structure. Using plasmid DNA of 4331 bp with varying polyamine concentration, NSPD was shown to induce strong inhibition on *in vitro* gene expression at high concentrations. AFM showed clear morphological differences in DNA treated with each polyamine, with NSPD thought to induce shrinkage with more potency through formation of smaller flower-like structures with multimolecular loops. The authors of this study also performed fluorescence microscopy, which correlated the effects on a higher-order structure, thereby supporting the features of ligand-DNA interaction as seen by AFM.

Beyond dsDNA, small molecules which target non-canonical DNA structures, such as G-quadruplex DNA, have shown promise as anti-cancer therapeutics. G-quadruplexes are secondary structures formed via the stacking of several planar layers that each consist of four Hoogsteen-bonded guanine residues.^216^ G-quadruplex forming sequences are abundant throughout the genome due to the high abundance of G-rich DNA regions, including oncogene promotors and telomeres.^217^ This, along with their ability to both interfere with nuclear machinery and promote replication fork stalling, creates attractiveness as anti-cancer therapeutic targets.^218^ Small molecules that can selectively stabilize these structures would promote DNA damage and drive genomic instability. G-quadruplex DNA has been observed by AFM to form large oligomeric G-wires,^153,155^ and short G-quadruplex structures sequences within DNA plasmids^154^ and minicircle DNA.^156^ AFM was used to characterize the interactions between G-quadruplex DNA with the G-quadruplex specific single-chain antibody HF1, and the G-quadruplex specific nuclear protein PARP-1. These experiments explicitly showed direct structural interactions.^219^ Furthermore, AFM was able to confirm the direct stabilizing effect of pyridostatin on transcription-produced G-quadruplexes as initially indicated by FRET melting experiments.^220^ To probe whether the stabilization kinetics of pyridostatin are affected by bending stress or topology Klejevskaja *et al.* used minicircle DNA containing G-quadruplexes.^156^ Klejevskaja *et al.* observed that G-quadruplex forming sequences behave differently within the topological constraint of a DNA minicircle, when compared to their behavior within linear DNA, or as isolated oligonucleotides. This study highlights another advantage of AFM in which larger, topologically complex constructs can be examined in solution, in real-time, and with molecular resolution.

As many chemotherapeutics influence DNA structure and function, AFM proves a useful tool in probing these interactions due to its high resolution and dynamic imaging capabilities. This has allowed for insights into binding characterization, structural and morphological changes, and stabilization kinetics between therapeutic ligands and DNA. Many of these studies have been conducted with established therapeutics currently used in clinical practice but illustrate how AFM may be a platform for investigating the interactions of therapeutic candidates that target DNA. With recent developments, these experiments could probe the topological dependence of ligands, the effects of intercalators on DNA molecules with intramolecular variations in groove depth, and the dynamics of structural rearrangements in real-time by exploiting the high temporal resolution of AFM.

## CONCLUSION

AFM has developed into an important technique within the sphere of biological imaging. The relatively simple sample preparation, coupled with the ability to image under physiological conditions and the possibility of dynamic imaging, provides AFM with a huge degree of versatility amongst conventional microscopy techniques. AFM imaging of DNA has validated structural and topological information once provided by established techniques and now provides new insights into how structural changes and flexibility affect enzymatic mechanics. With this understanding, the interplay between DNA and proteins has been investigated to better characterize these interactions and address challenges in understanding the role of DNA conformation in key biological processes. To that end, AFM also provides a unique perspective into visualizing the pharmacological mechanisms with which drugs are able to modulate these processes. This has been seen with established chemotherapeutics, which are known to influence DNA structure and function but may also prove useful in the pre-clinical characterization of therapeutic candidates. In this way, AFM may complement a host of other techniques involved in the development and optimization of therapeutics for an increasingly diverse clinical landscape.

## Data Availability

No new data were created or analyzed in this study.
